# CytoSorb hemoadsorption of apixaban during cardio-pulmonary bypass for heart transplantation

**DOI:** 10.1016/j.jhlto.2024.100165

**Published:** 2024-10-11

**Authors:** Anouk Frering, Antoine Abi Lutfallah, Aude Carillion, Daniel Wendt, Pascal Leprince, Adrien Bougle, Guillaume Lebreton

**Affiliations:** a Sorbonne University, Department of Cardiovascular and Thoracic Surgery, Institute of Cardiology, Pitié-Salpêtrière Hospital, Assistance Publique-Hôpitaux de Paris (AP-HP), Paris, France; b CytoSorbents Europe GmbH, Berlin, Germany; c Department of Thoracic- and Cardiovascular Surgery, Westgerman Heart and Vascular Center, University Duisburg-Essen, Essen, Germany

**Keywords:** heart transplantation, CytoSorb, direct oral anticoagulants, hemoadsorption, apixaban, cardio-pulmonary bypass

## Abstract

**Background:**

Heart transplantation is an emergency surgery requiring cardio-pulmonary bypass (CPB) and its timing is unpredictable. Patients on the transplant waiting list often have multiple reasons for being anticoagulated. Intraoperative removal of apixaban using CytoSorb seems to be an interesting solution for patients on DOACs requiring an emergency CPB intervention. The aim of this short communication is to describe the perioperative effects of the use of the CytoSorb hemoadsorption device during emergency CPB for a heart transplant patient.

**Methods:**

A 61-year-old male patient wait-listed for heart transplantation was admitted to our hospital to benefit from a heart transplantation. This patient, has an end-stage heart failure with multiple episodes of decompensation over the previous year. He was anticoagulated with a Vitamin K antagonist (VKA) due to atrial fibrillation and was switched to apixaban. Hemoadsorption by a CytoSorb cartridge was performed during the entire CPB duration. Anti-Factor Xa Activity (AFXaA) levels were taken before, during and after surgery in order to monitor anticoagulation.

**Results:**

Surgery consisted of an orthotopic heart transplantation with bi-caval anastomoses. At the time of anesthesia induction and after UFH administration, AFXaA levels were 330ng/mL and 317ng/mL, respectively. Thereafter, AFXaA decreased to 137ng/mL during CPB and to 57ng/mL after the end of CPB and protamine administration. After surgery, AFXaA levels stabilized over 50ng/mL over the next 14 hours. No primary graft dysfunction was observed, and during the post-operative period of 72 hours, the patient did not have any bleeding events requiring reintervention or transfusion.

**Conclusion:**

We observed that CytoSorb could be a potential solution to remove apixaban intraoperatively. If this efficacy is confirmed in larger trials, it would allow transplant candidates to be treated with DOACs without requiring a switch to VKAs.

## Background

Heart transplantation is an emergency surgery requiring cardio-pulmonary bypass (CPB) and its timing depends on the availability of a compatible graft. Patients on the transplant waiting list often have multiple reasons for being anticoagulated. Direct oral anticoagulants (DOACs) such as apixaban are very popular due to their well-known advantages, including fixed dosing, fewer drug and dietary interactions and no monitoring requirement. However, because of the graft's preservation time (usually limited to 4 hours in standard cold storage), and the half-life of apixaban (12 hours), this suggests that its clearance is not compatible with the transplantation time limit. Several strategies can be considered to manage anticoagulation. Prothrombin complex concentrate is commonly used as an effective alternative to reverse the effects of DOACs due to its rapid action on coagulation factors. Recently, a modified recombinant inactive form of human factor Xa (Andexanet alpha) developed for reversal of factor Xa inhibitors. Although, its potential interaction with unfractionated heparin (UFH) and widely its effects in cardiac surgery remains unknown, besides the fact that its high cost is a notable concern. Also, hemoadsorption devices can be an option to remove Apixaban from the bloodstream in cases of severe overdose or unexpected bleeding. Finally, in certain complex situations, a combination of these approaches may be warranted to optimize clinical management. Hemoadsorption using CytoSorb (CytoSorbents Inc., Princeton, NJ, USA) is a biocompatible sorbent bead-filled hemoperfusion cartridge, enabling the absorption of drugs, especially DOACs, which can easily be integrated into the CPB circuit. Therefore, intraoperative removal of apixaban seems to be an interesting solution for patients on antithrombotics requiring an emergency CPB intervention.

In this case report we describe the perioperative effects of the use of the CytoSorb hemoadsorption device during emergency CPB for a heart transplant patient.

## Case presentation

A 61-year-old male patient (75 kg/173 cm) wait-listed for heart transplantation was admitted to our hospital (Pitié-Salpêtrière Hospital, Sorbonne University, Paris, France) to benefit from a heart transplantation. His co-morbidities included systemic lupus erythematous with stage 3 chronic renal failure, hypertension, dyslipidemia, arteriopathy, and ischemic heart disease since 1997 with heart failure and multiple episodes of decompensation over the previous year. The patient was anticoagulated with a Vitamin K antagonist (VKA) due to atrial fibrillation and was switched to apixaban (5 mg twice daily) 9 days before surgery based on poor clotting control. The last dose of apixaban was taken 8 hours before surgery.

Transthoracic echocardiography showed a left ventricular ejection fraction of 15% with severe global hypokinesia, mitral regurgitation grade 3, tricuspid regurgitation grade 2, a non-dilated but hypokinetic right ventricle and a dilated non-compliant inferior vena cava. The risk for surgery as calculated by the EuroSCORE-II was 46.67%.

**Figure 1:**
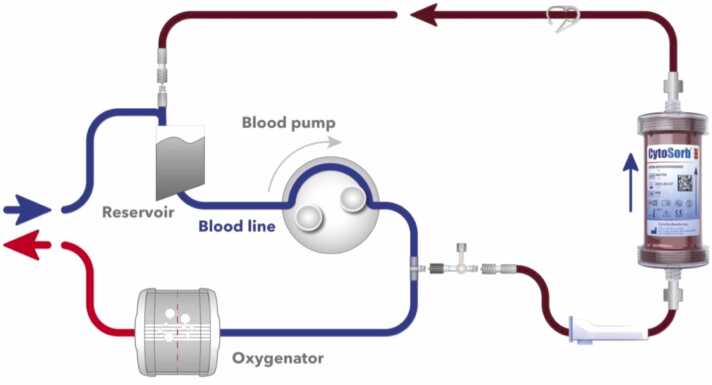
Installation of CytoSorb hemoadsorber on a cardiopulmonary bypass (CBP) circuit.

The CPB circuit was connected according to the standard protocol. A NaCl 0.9% primed CytoSorb cartridge was inserted between the oxygenator and the hard-shell reservoir. Hemoadsorption was performed during the entire CPB duration (Figure 1).

Anti-Factor Xa Activity (AFXaA) levels were taken at multiple time points before, during and after surgery in order to monitor anticoagulation.

## Results

Preoperatively, the patient's laboratory data showed moderate anemia (hemoglobin - Hb 11.4g/dl and hematocrit 33%), no thrombocytopenia (platelet count 209.10^9^/liter) and known chronic renal failure (serum creatinine 181 mmol/liter, glomerular filtration rate 35 ml/min/1.73m^2^). Activated Clotting Time (ACT) was 146 seconds.

The ACT reached 545 seconds after administration of 25,000 IU of UFH. Surgery consisted of an orthotopic heart transplantation with bi-caval anastomoses. CPB duration was 133 minutes with 83 minutes of aortic cross clamping. Total graft ischemic time was 249 minutes. After de-clamping, surgical hemostasis was completed by 2,000 IU of prothrombin complex concentrate, 2 packs of fresh frozen plasma, 1 pooled platelet concentrate, 4 packed red blood cells, 1.4 liter cell-saver and 2 g calcium gluconate, according to institutional policy to correct post–cardiopulmonary bypass coagulopathy guided by QUANTRA testing.

**Figure 2:**
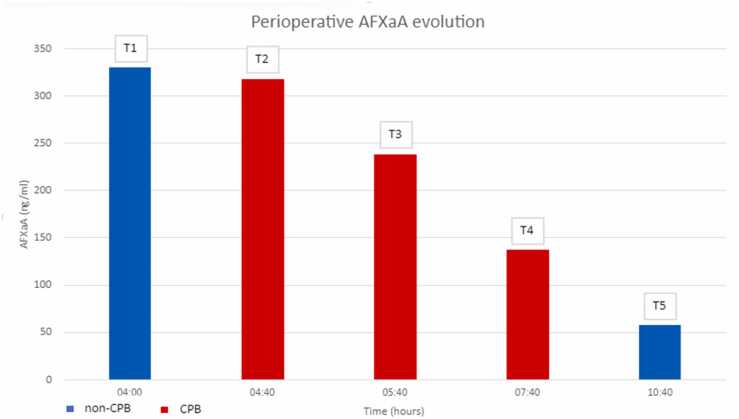
Perioperative plasma AFXaA levels (ng/ml). AFXaA, anti-factor Xa activity.

The perioperative evolution of plasma AFXaA levels are represented in Figure 2. At the time of anesthesia induction (T1) and after UFH administration (T2), AFXaA levels were 330 ng/ml and 317 ng/ml, respectively. Thereafter, AFXaA decreased to 137 ng/ml during CPB (T3) and to 57 ng/ml after the end of CPB and 250 mg (=25,000 IU) of protamine administration (T4).

After surgery, AFXaA levels stabilized over 50 ng/ml over the next 14 hours. Postoperative thromboprophylaxis was introduced 6 hours after surgery using UFH.

No primary graft dysfunction (PGD) was observed, and during the post-operative period of 72 hours, the patient did not have any bleeding events requiring reintervention or transfusion.

## Discussion

Patients on apixaban requiring non-deferrable on-pump cardiac surgery are at high risk of perioperative bleeding. In such emergent cases, especially in transplantation, the guidelines recommended DOAC-washout period of 48 hours is not possible. Many therapeutic options may be considered to mitigate the perioperative bleeding risk.^1^

Current guidelines^2^ regarding the perioperative management of DOACs, and specifically apixaban, suggest that in the case of emergent surgery, AFXaA should guide management: a concentration below the hemostatic safety threshold (30 ng/ml), no specific treatment is required. Beyond this threshold, the risk of bleeding increases, and if it exceeds 400 ng/ml, the bleeding risk is major. Specific (Andexanet alfa) or non-specific (prothrombin complex concentrates) reversal agents may therefore be administered.

The process of drug removal via an adsorption device may also be considered. Such cartridges can be easily integrated into various extracorporeal hemoperfusion circuits inlcuding cardiopulmonary bypass, extracorporeal membrane oxygenation (ECMO), continuous renal replacement therapy/continuous veno-venous hemofiltration, or simple hemoperfusion. CytoSorb is a CE-mark approved device, which consists of a cartridge filled with biocompatible divinylbenzene-coated sorbents, designed to remove ticagrelor and rivaroxaban during cardiopulmonary bypass assisted surgery. This proprietary technology uses size-selectivity to eliminate small hydrophobic molecules from blood, with removal efficiency being concentration-dependent. Each bead, ranging from 300 to 800 µm in size, possesses pores and channels, resulting in an effective surface area exceeding 40,000 m^2^ capable of binding hydrophobic small and medium-sized molecules.

This technique has already proven its effectiveness in reducing inflammatory cytokines and clearing other drugs.^3^, ^4^, ^5^ In the present case, intraoperative removal of apixaban, using the CytoSorb cartridge was analyzed by sequential measurements of AFXaA during heart transplantation. In another case report, Mendes et al.^6^ described similar results in a patient with apixaban admitted for emergent mitral valve replacement. Although the underlying indication in their experience was different, these 2 cases both used intraoperative hemoadsorption using Cytosorb for removal of apixaban. We observed a fast, effective and continuous removal of apixaban as measured by AFXaA.

At the end of CPB, the AFXaA was 50 ng/ml, compared to normal values of 30 ng/ml in patients without anticoagulant effects. Considering the quite high preoperative levels of AFXaA of 317 ng/ml, continuation of drug removal during the postoperative period should be discussed. Since the patient required neither ECMO or renal-dialysis and presented with no bleeding at the end of the procedure, we decided not to continue hemoadsorption post-operatively e.g. with a stand-alone hemoperfusion device.

Moreover, since AFXaA is a laboratory indicator these values are just a surrogate marker for perioperative bleeding events. Hassan et al^7^ investigated perioperative bleeding complications in patients on apixaban undergoing non-deferrable on-pump cardiac surgery, with or without intraoperative hemoadsorption. Hassan et al. have, to date, the largest evaluation of hemoadsorption for intraoperative apixaban removal and, compared to patients operated on without hemoadsorption, the bleeding complications were significantly lower in the CytoSorb group, which encourages us to hope that AFXaA provides a good estimate of this risk.

Currently, the STAR-D sham-controlled trial (NCT05093504) ongoing in the United States aims to evaluate the efficacy of DOAC removal during cardiopulmonary bypass for patients undergoing on-pump cardiothoracic surgery within 48 hours of last DOAC intake. The focus is on clinical endpoints like postoperative bleeding so these trial results are eagerly awaited, as they will help us to determine the effectiveness of antithrombotic reversal/removal strategies in patients undergoing non-deferrable surgery.

The potential impact of CytoSorb on the pharmacokinetic of concomitantly administered drugs must be considered, especially in anesthetic settings. To date, there is only 1 published report describing increased fentanyl requirements in COVID-19 patients on ECMO drugs in association with CytoSorb, whereas midazolam dosing appeared to be unaffected.^8^ Thus, real-time assessment of anesthetic drugs effect such as the use of relaxometry and bispectral index should be considered.

Of note, our patient presented with multiple comorbidities and received an organ that had undergone almost 4 hours of cold ischemia. It is interesting to note the absence of PGD, raising the question of a potential protective effect of hemoadsorption by CytoSorb, in line with the recent publication by Nemeth et al.^9^ Indeed, hemadsorption may reduce PGD by removing inflammatory mediators, such as interleukin-6 and tumor necrosis factor-alpha, which contribute to graft inflammation and dysfunction. Additionally, this technique could help eliminate toxins produced during ischemia-reperfusion injury, which could otherwise damage the graft. However, more extensive research is needed to confirm the efficacy of hemadsorption in reducing PGD and to optimize its clinical application.

In relation to our discussion on anticoagulation management in patients on Apixaban, the recent Direct Oral anticoagulant Therapy with the HeartMate 3 Study^10^ provides valuable insights. This study examined 8 Left Ventricular Assist Device patients bridged to heart transplantation while receiving Apixaban, using a protocol that included prothrombin complex concentrate and a cytokine adsorption cartridge. The results demonstrated successful discharge with preserved allograft function. However, increased use of blood products, temporary mechanical circulatory support, renal-replacement therapy, and longer hospital stays were observed, suggesting that caution is needed when using Apixaban as a bridging anticoagulation strategy. While a combined approach, including hemadsorption, may help control bleeding risks and improve outcomes, the long-term effects of this complex perioperative course on both patient and allograft outcomes remain uncertain and warrant further investigation.

## Informed consent

Informed consent was obtained from the patient for the publication of this case report and the use of anonymized clinical data.

## Conclusion

In conclusion, we observed that CytoSorb could be a potential solution to remove apixaban intraoperatively, although there is no certainty about its clinical efficacy with perioperative hemostasis management. If this efficacy is confirmed in larger trials, it would allow transplant candidates to be treated with DOACs without requiring a switch to VKAs.

## Conflicts of interest statement

Daniel Wendt reports a relationship with CytoSorbents Europe GmbH that includes: employment. One of the authors, Daniel Wendt, is employed by CytoSorbents Europe GmbH. This work did not receive any financial support or funding from CytoSorbents Europe GmbH. The authors declare that this employment relationship does not influence the design, execution, or interpretation of the research. If there are other authors, they declare that they have no known competing financial interests or personal relationships that could have appeared to influence the work reported in this paper.

## Glossary

ACT: Activated Clotting Time
AFXaA: Anti-Factor Xa Activity
CPB: Cardio-Pulmonary Bypass
DOACs: Direct Oral Anticoagulants
ECMO: Extracorporeal Membrane Oxygenation
LVEF: Left Ventricular Ejection Fraction
PGD: Primary Graft Dysfunction
UFH: Unfractionated Heparin
VKA: Vitamin K antagonist
